# Transcultural care provided by nurses to transgender women in primary health care: a qualitative study*

**DOI:** 10.1590/1518-8345.7760.4924

**Published:** 2026-10-16

**Authors:** Pedro Vitor Rocha Vila Nova, Glauber Weder dos Santos Silva, Arielly de Azevedo Candido, Raíssa Millena Silva Florencio, Glenda Roberta Oliveira Naiff Ferreira, Eliã Pinheiro Botelho

**Affiliations:** 1 Universidade Federal do Pará, Belém, PA, Brazil.; 2 Scholarship holder at the Coordenação de Aperfeiçoamento de Pessoal de Nível Superior (CAPES), Brazil.; 3 Secretaria de Estado da Saúde Pública do Rio Grande do Norte, Hospital Giselda Trigueiro, Natal, RN, Brazil.; 4 Universidade Federal do Pará, Faculdade de Enfermagem, Belém, PA, Brazil.; 5 Scholarship holder at the Conselho Nacional de Desenvolvimento Científico e Tecnológico (CNPq), Brazil.

**Keywords:** Transcultural Nursing, Transgender Persons, Vulnerable Populations, Sexual and Gender Minorities, Primary Health Care, National Health Strategies

## Abstract

**Objective:**

to explore the transcultural care provided by nurses to transgender women in primary health care.

**Method:**

qualitative, descriptive study based on Leininger’s Transcultural Care Theory. Ninety-four nurses working in primary health care across all regions of Brazil participated by completing an online form containing open-ended questions of a discursive nature. The data were analyzed using thematic content analysis, supported by IRaMuTeQ software.

**Results:**

four thematic categories emerged: challenges and barriers in health care; essential actions and care in primary health care; inclusion and respect in care; role of health professionals and community health workers.

**Conclusion:**

the results indicate that most nurses did not receive specific training or have protocols for caring for transgender women, revealing challenges related to prejudice, accessibility, and professional qualifications, but also initiatives for welcoming, inclusion, and action by community health workers.

## Highlights

**(1)** Lack of training hinders adequate care for transgender women.

**(2)** Cultural barriers and prejudice compromise universal and inclusive care.

**(3)** Nurses reinforce the importance of active listening and empathy in care.

**(4)** Continuing education and the provision of PrEP and PEP are essential for inclusion.

**(5)** Community Health Agents are crucial for the inclusion of transgender women.

## Introduction

In the context of Brazilian public policies, Primary Health Care (PHC) serves as the organizer and coordinator of care within the Health Care Network (RAS), with the Family Health Strategy (FHS) as the primary model for its strengthening and consolidation^1^.

When considering the multidisciplinary team comprising PHC, nurses stand out as essential professionals for the development of health promotion actions, as they act directly in the process of care, management, and implementation of the principles and guidelines of the Brazilian Unified Health System (SUS). Among their exclusive duties are conducting nursing consultations, planning, managing, coordinating, and evaluating the practices of nursing technicians and Community Health Agents (ACS)^2^.

In this study, the term “female nurse” was adopted to highlight the historical feminization of the profession in PHC, recognizing the presence of men to emphasize how care remains associated with the idea of “feminine nature”. This configuration reinforces gender stereotypes, makes work precarious, and contributes to the devaluation of the category^3^. Thus, the choice of terminology reflects the predominance of women and, at the same time, problematizes its effects, opening space for critical reflection on gender in nursing.

The strategic position of the female nurses in PHC directly contributes to changes in people‘s lifestyles, minimizing risk factors and promoting self-care capacity, making them protagonists in the care process^4^. In this context, it is essential to care for and welcome key and vulnerable populations, such as transgender women. This population faces various health inequalities on a daily basis, such as stigma and prejudice, violence, and difficulty accessing health services and other social goods. Institutional transphobia, discrimination, and disrespect for social names are among the main violations faced by transgender women^5^. These factors together contribute to the neglect of their health^6^ ^)-(^ ^7^.

The importance of implementing inclusive and competent healthcare is highlighted in the promotion of a welcoming and prejudice-free environment^8^. Based on the Theory of the Normalization Process, there is a need to train healthcare professionals in gender terminology and affirmative care practices to reduce barriers to access and improve the reception of care^9^. In addition, the main obstacles faced by transvestites and transsexuals are highlighted, such as inadequate reception and personal rejection by professionals^10^, which points to an urgent need for change in healthcare practices.

Given this context, a cross-cultural approach to caring for transgender women in PHC, which recognizes cultural particularities, is essential for nurses^11^. Continuing education and humanization of care are important for more inclusive and respectful practice^12^. The promotion of integrated care that considers the specificities of this population can contribute significantly to improving the health and well-being of transgender women, in addition to aligning with public health policies aimed at equitable access^13^.

Scientific production on nursing care, especially transcultural care, for transgender women in PHC, remains incipient and fragmented, with a small number of publications between 2008 and 2021. Existing studies indicate that structural and interpersonal stigmas, associated with the exclusion and fragmentation of health care for transvestites and transsexuals, still centered on specialized services, hinder the consolidation of the comprehensive model recommended by the SUS^14^ ^)-(^ ^15^. Barriers to access to PHC persist, such as disrespect for social name, in addition to the concentration of services and research in large urban centers, which limits a national vision and deepens regional inequalities^14^. Added to this is the inadequacy of initial and continuing training for nurses to deal culturally competently with the demands of this population^16^. These sets of evidence reveal the magnitude of the challenges faced in terms of gaps in training, practice, and health policies, justifying the relevance of the present study.

Given the scarcity and fragmentation of evidence on nursing care for transgender women in PHC, it is essential to describe and analyze its progress^17^. The cultural and social context of these women directly influences care practices, requiring sensitivity and cultural competence from professionals. Although public policies in Brazil have sought to consolidate a care network oriented towards equity, resoluteness, and comprehensiveness of care^18^ ^)-(^ ^19^, challenges remain in ensuring that such guidelines are implemented in PHC. Recognizing this cultural and social influence is therefore essential for guiding interventions that strengthen the quality and inclusion of care.

In this sense, this study aimed to explore the transcultural care provided by nurses to transgender women in primary health care.

## Method

### Type of study

This is a qualitative, descriptive study guided by the Consolidated Criteria for Reporting Qualitative Research (COREQ)^20^. This study is an excerpt from a quantitative-qualitative macro project involving 442 PHC nurses in different regions of Brazil, focusing, exclusively on the analysis of qualitative data in this article. The qualitative approach^21^ was chosen because it enables exploration of the personal and subjective experiences of PHC nurses to understand the cross-cultural care provided to transgender women, including how these experiences can impact professional practice.

Leininger’s Transcultural Care Theory^22^ was used as a theoretical framework to guide nursing practice by recognizing the diversity and universality of care, seeking cultural congruence between interventions and users’ values and beliefs, and developing cultural competence among professionals. Central to the theory is the axis of Culturally Competent (i.e., congruent), Care Action and Decision, operationalized in three ways: preservation/maintenance of beneficial cultural practices; accommodation/negotiation to adjust care to beliefs/rituals; and re-standardization/restructuring when changes are necessary for health improvements. In the study, the term Cultural Barrier and Accessibility refers to cultural/organizational obstacles that affect access to and continuity of care in PHC. These dimensions underpinned the organization and interpretation of the findings.

### Study setting

The study was conducted nationwide, carried out online, using a self-completed form, reaching nurses working in the FHS in all regions of Brazil, following the research eligibility criteria, without prior selection of specific health units.

In recent years, the ESF has expanded its coverage and consolidated its position as the primary model for strengthening PHC, thereby promoting the reorganization of the RAS. Due to Brazil’s vast territory and regional differences, FHS coverage varies between regions, states, and municipalities. The Northeast region has the highest FHS coverage, at 82.0%, followed by the Midwest and North regions, both at 65.0%, the South at 63.7%, and the Southeast at 51.0%.

### Period

The data was collected between August and December 2023.

### Population

The population consisted of nurses working in PHC and members of FHS teams across all regions of Brazil, who were directly engaged in healthcare.

### Selection criteria

As inclusion criteria, participants with at least six months of experience in the FHS, who had already provided nursing consultations to transgender women were selected. The six-month time frame was adopted to ensure that participants had minimal experience with the PHC routine and familiarity with the service’s dynamics, thereby qualifying their reports. Participants who were on leave or vacation during the data collection period were excluded.

### Sample definition

For this qualitative analysis, all 94 nurses working in the FHS, which were selected via convenience sampling and completed an open-ended questionnaire via an online form, the average completion time was 25 minutes. Thus, the sample included participants from all regions of the country, without the intention of ensuring national representativeness, but with consideration for the geographical diversity of the respondents.

### Instrument and data collection 

Data were collected via a quantitative-qualitative online form, available on the Google Forms platform, which allows only one submission per Internet Protocol (IP) address, ensuring greater security and preventing duplication. Prior contact and the link to the form were distributed to nurses via instant messaging (WhatsApp), social media (Instagram), or email. There was no field note as this was an online survey.

The specific macroproject form contained 31 closed-ended questions (multiple-choice) and two open-ended questions of a discursive nature, which supported the qualitative analysis of this study. For the present study, variables from the closed questions were used only to characterize the sociodemographic (age, gender identity), occupational (length of service in the FHS, number of employment contracts, length of training, remuneration), and academic (education and qualifications) profiles of the participants. The open-ended questions addressed experiences in caring for transgender women in PHC, namely: describe your care and provision of care to transgender women in your reality in the FHS; what challenges and needs do you identify in caring for transgender women in PHC? The questions were previously tested and validated by the research group before administration. 

To conduct the research, 10 data collectors (five undergraduates and five master’s students) were selected from the five macro-regions of Brazil. They underwent specific training to identify and invite potential participants, as well as to support the dissemination and management of access to online instruments [collection form and Free and Informed Consent Form (FICF)].

The lead author, a master’s student and cisgender man, served as the data collection coordinator and participated in an eight-hour training session given by a postdoctoral researcher with extensive experience in qualitative research. The training was conducted in an expository-dialogical manner, with a critical-reflective character, as well addressed the characterization of qualitative research, the importance of reflexivity, and strategies for approaching and enrolling participants. 

At the time of the study, the research team consisted of one undergraduate, one master’s student, three PhD professors, and one postdoctoral researcher, all members of a study group on sexually transmitted infections (STI) in the Amazonian context, with an interest in vulnerable populations.

Data collection for the macro project was carried out by disseminating the form online and, additionally, by directly approaching potential participants. The data collection coordinator established prior contact with nurses to present the study’s objectives and rationale, thereby strengthening the relationship of trust. In addition, data collectors contacted professionals in their regions and localities, who, in turn, referred to other eligible nurses. This procedure, like the snowball sampling technique, was adopted to broaden the study’s scope and include participants who might not have been reached through online dissemination alone.

### Data processing and analysis

Qualitative data were processed using the software *Interface de R pour les Analyses Multidimensionnelles de Textes et de Questionnaires* (IRaMuTeQ), and the analysis was discussed among the authors. Among the textual analysis options, we opted for Descending Hierarchical Classification (DHC), proposed by Reinert^23^. This technique classifies text segments based on their own vocabularies, subdividing them according to the frequency of forms. The elements included in their respective classes were those with a frequency ≥ 40 occurrences in the corpus and association with the class determined by a χ² value ≥ 3.98, considering a 95% confidence interval and a significance level of 5% (*p*< 0.05). DHC performs semantic processing and identifies the context in which words are used.

Subsequently, content analysis with thematic formulation was performed, based on the processing of the *corpus* in IRaMuTeQ using DHC, which generated the class structure and served as the initial tree for thematic analysis. This procedure enabled initial coding by semantically classifying the terms into categories, a step analogous to the coding process of content analysis. Next, the stages of material exploration, treatment of results, inference, and interpretation were conducted using thematic content analysis^24^.

As this was a self-completed, anonymous *online* form, no feedback was given to participants. Furthermore, achieving theoretical saturation was not the main objective of this study, since it is part of a larger project; however, the sample was considered sufficient to consolidate the categories and meet the qualitative objective. Similarly, the interpretations were not returned to the participants, and the analysis was limited to the researchers’ interpretation.

### Ethical aspects

The broader project that gave rise to this article was approved by the Research Ethics Committee of the Institute of Health Sciences of the Federal University of Pará (Opinion No. 5,966,169 of March 27, 2023, CAAE 66895423.0.0000.0018). The original research employed both a quantitative and qualitative approach and had three specific objectives. The present study corresponds to a section of this investigation, referring to the third specific objective, which addresses the qualitative stage exclusively, based on the analysis of open-ended responses in the collection form. All participants signed the informed consent form.

To ensure anonymity, the statements were identified using alphanumeric codes. We chose to maintain the original numbering assigned in the macro project, so that the numerical sequence does not correspond to the total number of participants in this sample (n=94), but to the larger set of respondents (n=442). This strategy aimed to preserve the database’s standardization and ensure analytical traceability, in line with the ethical principles of qualitative research and the recommendations of COREQ^20^.

## Results

Throughout the text, the term nurse was used in the feminine form as a terminological option, given that, among the 94 participants, 79 (84.0%) self-identified as female. Among them, 48 (51.0%) were older than 35 years. The respondents were distributed across the five regions of Brazil: North (n=37; 39.4%), Northeast (n=15; 16.0%), Midwest (n=six; 6.4%), South (n=10; 10.6%), and Southeast (n=26; 27.6%).

The nurses had specializations in Public Health related to PHC (n=52, 55.3%) or Master‘s/Doctorate degrees (n=17, 18.1%). Most had never received specific training in caring for transgender women (n=52, 55.3%) and did not adopt care protocols for transgender women in their health service (n=75, 79.8%).

In terms of professional aspects, they had completed their nursing degree less than 10 years ago (n=60, 63.8%), and had been working in the FHS for less than 10 years (n=66, 70.2%), had only one employment relationship (n=71, 77.6%) and earned less than five minimum wages (minimum wage in Brazil in 2023: BRL 1,320.00) (n=61, 64.9%).

 Processing the corpus (54 pages), using DHC revealed 6,603 occurrences of terms, presented in 1,478 distinct forms, across 94 text segments, with each paragraph treated as a single text segment. The analysis retained 75.5% of the corpus, which was considered satisfactory. The objective of this processing was to identify indicators of meaning in the participants’ responses. DHC generated four semantic classes (Figure 1) and grouped together terms that were associated with one another and used by participants to assign meaning to the objects of discussion.

**Figure 1:** Classes included in the dendrogram generated by descending hierarchical classification in IRaMuTeQ software. Belém, PA, Brazil

*Corpus*	Classes and topics	Postulates of Transcultural Care Theory	Terms (f*/x2^†^)
**“Nurses_trans_women_FHS”**	**Class 1**	Diversity and Universality of Care	Transgender (100/14.57), population (50/13.77), understand (100/10.77), primary health care (50/7.54), training (50/7.74), respect (66.67/7.31), difficulty (46.15/5.09), prejudice (40/4.86), network (83.33/16.75), qualified (60/4.32), lack (66.67/5.51).
	Challenges and barriers in healthcare	Cultural Barriers and Accessibility	
	**Class 2**	Cultural Congruence in Care	Group (77.78/21.95), provide (70/18.59), action (83.33/16.75), public (44/14.37), care (44.44/9.31), promote (75/8.18), need (60.67/4.36), offer (60/5.53), procedure (66.67/4.36), awareness (66.67/4.36), assistance (44.44/3.98).
	Essential actions and care in Primary Health Care		
	**Class 3**	Culturally Competent Care Action and Decision Making	User (77.78/11.5), distinction (100/10.09), outpatient clinic (80.33/9.09), care (45.71/8.63), respect (100/7.46), PrEP^‡^ (80/6.57), PEP§ (75/4.2), training (60/5.17), women (62.5/4.69), inclusion (66.67/4.33).
	Inclusion and respect in customer service		
	**Class 4**	Cultural Competence	Community health worker (90/21.99), doctor (100/13.72), bring (100/13.72), visit (100/7.99), home (100/7.99), accept (100/7.99), nursing (71.43/7.18), active (80/7.14), consultation (62.5/5.25), patient (53.85/5.19), main (66.67/4.80), independent (75/4.59).
	Role of healthcare professionals and Community Health Workers		

*f = Frequency, ^†^x^2^ = Chi-square test value, ^‡^PrEP = Pre-exposure prophylaxis, ^§^PEP = Post-exposure prophylaxis

The classes were organized into thematic categories and interpreted in accordance with Leininger’s Transcultural Care Theory^22^.

### Thematic class 1 - Challenges and Barriers in Health Care

The diversity and universality of care are reflected in the need to adapt health services to meet the different needs of transgender women. The universality of care is reinforced by empathy and acceptance, whereas diversity manifests in specific barriers, such as prejudice and stigma. *Pure prejudice. This issue is not addressed by technical references on women’s health. [...] PHC is the gateway to welcoming and preventive health care* (Nurse_12)*. Prejudice, lack of information, and unpreparedness of health professionals in their relationship with transgender people* (Nurse_45). *Stigma among other professionals working in the unit regarding how to approach this population and provide adequate care* (Nurse_39). *Prejudice, lack of information, and unpreparedness of health professionals in their relationship with transgender people* (Nurse_404). *The main challenge is for transgender women to accept themselves; they usually suffer a lot of prejudice and because of this, it makes it difficult to provide care* (Nurse_282).

Cultural barriers and accessibility issues are highlighted by the challenges of providing care. Accessibility is compromised by insufficient professional preparation and the absence of specific protocols. *Lack of training and defined workflow for caring for transgender* people (Nurse_92). *There is no training for caring for these transgender women, a lack of knowledge about care workflows [...]* (Nurse_76). *I do not observe a clinical protocol to guide care* (Nurse_45). *The lack of knowledge about the network that could assist* with certain processes for these patients, and *the lack of technical training in caring for this population [...]* (Nurse_204).

### Thematic class 2 - Essential Actions and Care in Primary Health Care

Cultural congruence in care is reflected in the provision of services and the promotion of a welcoming and empathetic environment. Active listening and personalized care are essential to ensure culturally congruent care. *I offer group activities, conduct examinations, and request follow-ups. I strive to project empathy, humanization, and guidance on healthcare across its various dimensions-physical, psychological, biological, and social* (Nurse_107). *Welcoming, effective listening, bonding, offering services at the primary level and in all services* (Nurse_282)*. I provide comprehensive care, without distinction* (Nurse_421*). I am responsible for the STI line, I belong to the LGBT community* [Lesbian, Gay, Bisexual, and Transgender]*, I listen with empathy, I take a complete medical history, I break the ice and stigma, I promote a welcoming environment, and I offer all available* services (Nurse_305*).*

### Thematic class 3 - Inclusion and Respect in Care

The lack of training for health professionals was highlighted as a central challenge in combating prejudice and improving the quality of care for transgender women. *Lack of training on how to care for this population* (Nurse_09). *Lack of training in caring for transgender women, difficulty for this population in accessing quality healthcare, and lack of qualified and compassionate professionals to care for them [...]* (Nurse_25). *Lack of management incentive, lack of training, and difficulty in getting transgender women to participate in the unit’s activities. I have the appropriate professional profile, I am a cis gay man, I have professional qualifications, skills, and experience in my daily life with the issue* (Nurse_30). *Technical training and provision of services with a view to welcoming the patient* (Nurse_21). *Ongoing training to avoid prejudice and improve care* (Nurse_34). *We need specialized training to learn the best way to welcome and approach transgender women. Having a specific care flow that we can use to meet this demand (*Nurse_194*). That transgender women come to PHC more often, that professionals understand and respect them, that training for care is increased [...] and that a protocol for care without barriers* is created (Nurse_384*).*

However, culturally competent care is evident in the inclusion of specific practices, such as PrEP and PEP, and in ongoing professional training. Cultural competence is essential to providing respectful and inclusive care. *Qualification, access to PrEP and PEP, specific HPV [Human Papillomavirus] collection material, internal waiting room* (Nurse_300). *Lack of training and defined workflow for caring for transgender* people (Nurse_457). *All professionals need training focused on transgender* people (Nurse_425).

### Thematic class 4 - Role of Health Professionals and Community Health Workers

Cultural competence is evident in the importance of CHA and other health professionals in establishing appropriate community ties and care. *After extensive work, we have contact and home visits from CHA, nurses, and doctors, and we have lectures in schools and at the FHS, so that we can be closer* (Nurse_45). *Starting care in PHC through the FHS makes access easier, even because we can count on the work of CHA, who identify individuals who are distant from health services, thus enabling active search and their inclusion in health care* (Nurse_70). *An adequate number of CHA to bring these patients to the unit* (Nurse_19).

The willingness of nurses to learn and adapt to the needs of transgender women demonstrates culturally competent care. *Respecting gender identity and using the correct pronouns when providing care* (Nurse_1). *Adapting protocols to be more inclusive* (Nurse_28). *The CHA is our bridge, as soon as they identify someone, they take a humanized approach, bringing that woman to the unit where the nursing consultation takes place (*Nurse_101*).*

## Discussion

This study sought to explore the transcultural care provided by PHC nurses to transgender women, with an emphasis on their work within the Family Health Program (FHS). This population has specific health needs resulting from the culture they develop as an identity group and the heteronormative violence they face for questioning imposed gender norms. Leininger’s Transcultural Care Theory^22^ guided the interpretation of the findings by revealing the coexistence of diversity and universality in care, the search for cultural congruence in the personalization of actions, and the centrality of cultural competence in sustaining inclusive practices.

The universality of care in PHC is a fundamental principle for meeting the needs of transgender women. However, challenges remain that compromise the implementation of this principle, such as insufficient training and prejudice among health professionals, which compromise the practice of transcultural care. The notes taken by participating nurses also show a lack of specific information and protocols, resulting in inappropriate approaches that perpetuate stigma and hinder access to health services^25^ ^)-(^ ^26^.

In terms of specific needs, the most notable demands are for hormone therapy, access to PrEP/PEP prophylaxis, adequate materials and procedures for collecting tests, use of social names and recognition of gender identity, as well as a welcoming environment with qualified listening. The lack of technical preparation and clear guidelines for these fronts was reiterated, revealing gaps in continuing education and institutional support^27^.

The findings suggest that these gaps are not merely technical: they reflect structural gender inequalities that permeate healthcare organizations. Overcoming them requires not only specific qualifications, but also broader organizational and cultural changes, such as committed management, accountability, and monitoring. The lack of institutional support and incentives for professional qualification results in low adherence of transgender women to health unit activities, indicating the urgent need for leadership commitment to promoting training and awareness to transform these challenges into opportunities for improvement.

Despite these barriers, cultural competence is an essential pillar of healthcare practice. The inclusion of specific practices, such as the use of PrEP and PEP, as well as the continuous training of professionals, is fundamental to providing care that respects and includes the specificities of transgender women^12^ ^),(^ ^28^. In addition, research has shown that personalizing care through active listening and welcoming attitudes is crucial to ensuring that transgender women feel safe and respected during care, promoting a positive experience in the search for care^29^.

The national scope of the study, covering different regions, also reveals potential regional asymmetries in cross-cultural care. While some locations have developed practices of welcoming and inclusion, others continue to face significant barriers to access and professional unpreparedness, reflecting unequal coverage of the FHS and distinct cultural contexts. This heterogeneity underscores the challenge of conceptualizing cross-cultural care in a multicultural country and of adapting interventions to local realities.

The formation of bonds between health professionals and the community is considered a determinant of the success of care provided. The role of CHAs is particularly emphasized, as they are at the forefront of community contact and play a vital role in identifying and including people who may be distant from health services^30^ ^)-(^ ^31^. Research^32^ has shown that strategies such as home visits and school-based lectures are effective in bringing health services closer to the community, thereby making care more accessible and humanized.

Nursing practice should be based on empathy, acceptance, and personalized care, recognizing and valuing the specificities of transgender women. Primary care services should be comprehensive and accessible, ensuring that people have access to all available services and guaranteeing continuous, high-quality care^28^ ^)-(^ ^29^ ^),(^ ^33^. This implies a commitment by health professionals to treat all people with dignity and respect, promoting a health system that is inclusive and sensitive to cultural and social diversity.

The implementation of adapted protocols and ongoing training of professionals are essential to ensure comprehensive health care for transgender women. The adaptation and operationalization of these protocols should reflect an institutional commitment to equity, involving leadership, management, and the engagement of professionals to translate recommendations into effective practices^34^ ^)-(^ ^36^. Thus, ongoing training is a key element of care transformation, as it prepares professionals to address the specificities of caring for this population.

The findings of this study have important implications for health and nursing. They highlight the need for ongoing training of nurses in primary health care for the care of transgender women, as well as inclusive protocols that guide practices sensitive to gender diversity. They also reinforce the central role of nurses and community health workers in expanding access, strengthening bonds, and promoting a stigma-free care environment. By emphasizing cultural competence as the foundation of practice, the study contributes to improving health care and developing more equitable and inclusive services.

However, this study has methodological limitations. The findings specifically reflect the perceptions of PHC nurses, especially those working in the FHS, so that possible selection bias restricts its transferability to other contexts of nursing practice with transgender women. Furthermore, the study focused on the perceptions of these professionals, without directly including the voices of users, whose participation in future research could broaden the understanding of care in PHC. Other dissident populations, such as transgender men and non-binary people, were also not included, requiring their own studies^17^.

In summary, cultural competence and the active role of FHS professionals, especially nurses, are fundamental to the cross-cultural care provided to transgender women in PHC. These practices not only improve access to health services but also promote a more inclusive, respectful, and effective care environment that meets the unique needs of this population. Continuing education and the implementation of public policies that ensure professional training are essential to advancing scientific knowledge and healthcare practice.

## Conclusion

In the qualitative sample of nurses working in the FHS, the findings show that care for transgender women in PHC advances when there is qualified listening, welcoming, and personalization of actions, with community mediation by CHA; and it is weakened in the face of stigma, lack of protocols, and training gaps, resulting in difficult access and practices that are insensitive to the specificities of this population. Anchored in Transcultural Care Theory, the interpretation reveals that cultural competence is not only an individual attribute but also a function of organizational arrangements; its deficiency helps explain the observed barriers, including regional heterogeneities within Brazilian PHC.

For nursing practice, the results imply continuing education in cultural competence, adaptation, and operationalization of inclusive protocols (clear lines of care and flows; minimum indicators such as recording social names and offering/referring to PrEP/PEP), health matrix support, combating misinformation, and the participation of transgender people in the co-construction of care processes. Strengthening the role of CHA in active search and mediation with the territory, integration with specialized services, and leadership commitment to equity and continuous monitoring are viable approaches within the daily routine of PHC to translate recommendations into effective practices, thereby expanding access, quality, and safety of care.

## Data Availability

All data generated or analysed during this study are included in this published article.
