# Supplementary material for: Transcultural care provided by nurses to transgender women in primary health care: a qualitative study
Source: Rev Lat Am Enfermagem. 2026 Oct 16;34:e4924. doi: 10.1590/1518-8345.7760.4924 (PMC13674968; doi:10.1590/1518-8345.7760.4924)
Supplement: Translation

# Translation: Cuidado transcultural das enfermeiras prestado a mulheres transexuais na atenção primária à saúde: estudo qualitativo*

**DOI:** 10.1590/1518-8345.7760.4925

**Authors:** Pedro Vitor Rocha Vila Nova, Glauber Weder dos Santos Silva, Arielly de Azevedo Candido, Raíssa Millena Silva Florencio, Glenda Roberta Oliveira Naiff Ferreira, Eliã Pinheiro Botelho

**Affiliations:** 1 Universidade Federal do Pará, Belém, PA, Brasil.; 2 Bolsista da Coordenação de Aperfeiçoamento de Pessoal de Nível Superior (CAPES), Brasil.; 3 Secretaria de Estado da Saúde Pública do Rio Grande do Norte, Hospital Giselda Trigueiro, Natal, RN, Brasil.; 4 Universidade Federal do Pará, Faculdade de Enfermagem, Belém, PA, Brasil.; 5 Bolsista do Conselho Nacional de Desenvolvimento Científico e Tecnológico (CNPq), Brasil.

**Keywords:** Enfermagem Transcultural, Pessoas Transgênero, Populações Vulneráveis, Minorias Sexuais e de Gênero, Atenção Primária à Saúde, Estratégias de Saúde Nacionais

## Abstract

**Objetivo:**

explorar o cuidado transcultural das enfermeiras prestado a mulheres transexuais na atenção primária à saúde.

**Método:**

estudo qualitativo, do tipo descritivo, ancorado na Teoria do Cuidado Transcultural de Leininger. Participaram 94 enfermeiras da atenção primária à saúde de todas as regiões do Brasil, por meio de autopreenchimento de formulário *online* contendo perguntas abertas de natureza discursiva. Os dados foram analisados pela análise de conteúdo temática, com suporte do *software* IRaMuTeQ.

**Resultados:**

emergiram quatro categorias temáticas: desafios e barreiras no atendimento de saúde; ações e cuidados essenciais na atenção primária à saúde; inclusão e respeito no atendimento; papel dos profissionais de saúde e do agente comunitário de saúde.

**Conclusão:**

os resultados indicam que a maioria das enfermeiras não recebeu treinamento específico nem dispunha de protocolos para o cuidado a mulheres transexuais, desvelando desafios relacionados ao preconceito, à acessibilidade e à qualificação profissional, mas também iniciativas de acolhimento, inclusão e atuação dos agentes comunitários de saúde.

## Destaques

**(1)** Falta de qualificação dificulta o atendimento adequado às mulheres transexuais.

**(2)** Barreiras culturais e preconceito comprometem o cuidado universal e inclusivo.

**(3)** Enfermeiras areforçam a importância da escuta ativa e empatia no atendimento.

**(4)** Capacitação contínua e oferta de PrEP e PEP são essenciais para inclusão.

**(5)** Agentes Comunitários de Saúde são cruciais para a inclusão das mulheres transexuais.

## Introdução

No contexto das políticas públicas brasileiras, a Atenção Primária à Saúde (APS) configura-se como ordenadora e coordenadora do cuidado no âmbito da Rede de Atenção à Saúde (RAS), sendo a Estratégia Saúde da Família (ESF) o principal modelo para seu fortalecimento e consolidação^1^.

Ao se considerar a equipe multiprofissional que integra a APS, a enfermeira destaca-se como profissional essencial para o desenvolvimento das ações de promoção da saúde, atuando diretamente no processo de cuidado, gestão e implementação dos princípios e diretrizes do Sistema Único de Saúde (SUS). Entre suas atribuições privativas estão a realização da consulta de enfermagem, o planejamento, o gerenciamento, a coordenação e a avaliação das práticas dos técnicos de enfermagem e dos Agentes Comunitários de Saúde (ACS)^2^.

Neste estudo, adotou-se o termo “enfermeira” para destacar a feminização histórica da profissão na APS, reconhecendo a presença de homens, mas enfatizando como o cuidado segue associado à ideia de “natureza feminina”. Essa configuração reforça estereótipos de gênero, precariza o trabalho e contribui para a desvalorização da categoria^3^. Assim, a escolha terminológica reflete a predominância feminina e, ao mesmo tempo, problematiza seus efeitos, abrindo espaço à reflexão crítica sobre gênero na enfermagem. 

A posição estratégica da enfermeira na APS contribui diretamente para mudanças no estilo de vida das pessoas, minimizando os fatores de risco e promovendo a capacidade de autocuidado, tornando-as protagonistas no processo de cuidado^4^. Nesse contexto, é imprescindível o atendimento e acolhimento de populações-chave e vulnerabilizadas, como as mulheres transexuais. Essa população convive, diariamente, com diversas iniquidades em saúde, como estigmas e preconceitos, violências, dificuldade de acesso aos serviços de saúde e outros bens sociais. A transfobia institucional, a discriminação e o desrespeito ao nome social estão entre as principais violações enfrentadas por mulheres transexuais^5^. Esses fatores, em conjunto, contribuem para que sua saúde seja negligenciada^6^ ^)-(^ ^7^ ^).^

A importância de implementar cuidados de saúde inclusivos e competentes destaca-se na promoção de um ambiente acolhedor e livre de preconceitos^8^. Com base na Teoria do Processo de Normalização, observa-se a necessidade de qualificar profissionais de saúde em terminologia de gênero e práticas de cuidado afirmativo para reduzir barreiras de acesso e melhorar o acolhimento no atendimento^9^. Além disso, são evidenciados os principais obstáculos enfrentados por pessoas travestis e transexuais, como o acolhimento inadequado e a rejeição pessoal por parte dos profissionais^10^, o que aponta para uma mudança urgente nas práticas de saúde.

Diante desse contexto, uma abordagem transcultural no atendimento às mulheres transexuais na APS, que reconheça as particularidades culturais, mostra-se imprescindível para a enfermeira^11^. A importância de uma formação contínua e da humanização do atendimento é importante para uma prática mais inclusiva e respeitosa^12^. A promoção de um cuidado integrado, que considere as especificidades dessa população, pode contribuir significativamente para a melhoria da saúde e bem-estar das mulheres transexuais, além de alinhar-se com as políticas públicas de saúde voltadas para a equidade no acesso^13^. 

A produção científica sobre os cuidados de enfermagem, especialmente transculturais, destinados às mulheres transexuais na APS, permanece incipiente e fragmentada, com número reduzido de publicações no período de 2008 a 2021. Os estudos existentes apontam que estigmas estruturais e interpessoais, associados à exclusão e fragmentação da atenção à saúde de travestis e transexuais, ainda centrada em serviços especializados, dificultam a consolidação do modelo integral preconizado pelo SUS^14^ ^)-(^ ^15^. Persistem barreiras no acesso à APS, como o desrespeito ao nome social, além da concentração de serviço e pesquisas em grandes centros urbanos, o que limita uma visão nacional e aprofunda desigualdades regionais^14^. Soma-se a isso a insuficiência da formação inicial e contínua de enfermeiras para lidar de forma culturalmente competente com as demandas dessa população^16^. Esses conjuntos de evidências revelam a magnitude dos desafios enfrentados quanto às lacunas na formação, na prática e nas políticas de saúde, justificando a relevância do presente estudo.

Diante da escassez e fragmentação das evidências sobre o cuidado de enfermagem às mulheres transexuais na APS, torna-se fundamental descrever e analisar seu avanço^17^. O contexto cultural e social dessas mulheres influencia diretamente as práticas assistenciais, demandando sensibilidade e competência cultural dos profissionais. Embora políticas públicas no Brasil tenham buscado consolidar uma rede de atenção orientada para a equidade, a resolutividade e a integralidade do cuidado^18^ ^)-(^ ^19^, persistem desafios para garantir que tais diretrizes se concretizem na APS. Reconhecer essa influência cultural e social é, portanto, essencial para orientar intervenções que fortaleçam a qualidade e a inclusão no atendimento.

Neste sentido, este estudo teve como objetivo explorar o cuidado transcultural das enfermeiras prestado a mulheres transexuais na atenção primária à saúde.

## Método

### Tipo de estudo

Trata-se de estudo qualitativo, descritivo, orientado pelas diretrizes do *Consolidated Criteria for Reporting Qualitative Research* (COREQ)^20^. Este estudo é um recorte de um macroprojeto quanti-qualitativo que contou com a participação de 442 enfermeiras da APS em diferentes regiões do Brasil, detendo-se, neste artigo, exclusivamente na análise dos dados qualitativos. A abordagem qualitativa^21^ foi escolhida por permitir explorar as experiências pessoais e subjetivas de enfermeiras da APS para fins de compreender o cuidado transcultural prestado a mulheres transexuais, incluindo o modo como tais vivências podem produzir impactos na prática profissional.

A Teoria do Cuidado Transcultural de Leininger^22^ foi utilizada como referencial teórico e orienta a prática de enfermagem a partir do reconhecimento da diversidade e universalidade do cuidado, da busca por congruência cultural entre intervenções e valores/credores do usuário e do desenvolvimento de competência cultural pelas profissionais. Central à teoria está o eixo de Ação e Decisão de Cuidados Culturalmente Competentes (isto é, congruentes), operacionalizado em três modos: preservação/manutenção de práticas culturais benéficas; acomodação/negociação para ajustar o cuidado às crenças/rituais; e repadronização/reestruturação quando mudanças são necessárias para melhoras em saúde. No estudo, o termo Barreira Cultural e Acessibilidade designa obstáculos de ordem cultural/organizacional que incidem sobre o acesso e a continuidade do cuidado na APS. Essas dimensões sustentaram a organização e a interpretação dos achados.

### Cenário do estudo

O estudo foi realizado em âmbito nacional, conduzido de forma *online*, por meio de formulário autopreenchido, alcançando enfermeiras atuantes na ESF de todas as regiões do Brasil, seguindo critérios de elegibilidade da pesquisa, sem a seleção prévia de unidades de saúde específicas.

Nos últimos anos, a ESF tem expandido sua cobertura e se consolidado como o principal modelo para o fortalecimento da APS, promovendo a reorganização da RAS. Devido ao vasto território brasileiro e às diferenças regionais, a cobertura da ESF varia entre regiões, estados e municípios. A região Nordeste apresenta a maior cobertura de ESF, com 82,0%, seguida pelas regiões Centro-Oeste e Norte, ambas com 65,0%, Sul com 63,7%, e Sudeste com 51,0%.

### Período

Os dados foram coletados no período entre agosto e dezembro de 2023.

### População

A população foi composta por enfermeiras e enfermeiros que trabalhavam na APS, integrantes das equipes da ESF em todas as regiões do Brasil, atuando diretamente na assistência.

### Critérios de seleção

Como critério de inclusão, foram selecionados participantes com pelo menos seis meses de atuação na ESF, e que já tinham realizado consulta de enfermagem para mulheres transexuais. O recorte temporal de seis meses foi adotado para assegurar que as participantes tivessem experiência mínima de inserção na rotina da APS e familiaridade com a dinâmica do serviço, de modo a qualificar seus relatos. Foram excluídas participantes que estavam em algum tipo de licença trabalhista ou de férias durante o período de coleta de dados.

### Definição da amostra

Para esta análise qualitativa, foi selecionada por conveniência a totalidade das 94 enfermeiras que atuavam na ESF, as quais responderam às questões abertas do formulário online autopreenchido, cujo tempo médio de preenchimento foi de 25 minutos. Assim, a amostra incluiu participantes de todas as regiões do país, sem a pretensão de assegurar representatividade nacional, mas contemplando a diversidade geográfica das respondentes.

### Instrumento de coleta dos dados

Os dados foram coletados por meio do autopreenchimento de um formulário *online* quanti-qualitativo, disponibilizado na plataforma Google *Forms*, que permite somente uma submissão por *Internet Protocol* (IP), garantindo maior segurança e evitando duplicidades. O contato prévio e o link do formulário foram distribuídos às enfermeiras por meio de mensageiro eletrônico (WhatsApp), rede social (Instagram) ou e-mail. Não houve nota de campo por tratar-se de pesquisa *online*.

O formulário específico do macroprojeto continha 31 questões fechadas (múltipla escolha) e duas questões abertas de natureza discursiva, que subsidiaram a análise qualitativa deste estudo. Para o presente recorte, das questões fechadas, foram utilizadas variáveis apenas para caracterizar o perfil sociodemográfico (idade, identidade de gênero), laboral (tempo de atuação na ESF, quantidade de vínculos, tempo de formação, remuneração) e acadêmico (formação e qualificação) das participantes. As questões abertas abordavam as experiências no atendimento às mulheres transexuais na APS, a saber: descreva seu atendimento e prestação de cuidados às mulheres transexuais na sua realidade na ESF; quais desafios e necessidades você identifica no atendimento às mulheres transexuais na APS? As perguntas foram testadas previamente e validadas pelo grupo de pesquisa antes de sua aplicação. 

Para a condução da pesquisa, foram selecionados 10 coletores de dados (cinco estudantes de graduação e cinco de mestrado), provenientes das cinco macrorregiões do Brasil, os quais passaram por treinamento específico para identificar e convidar potenciais participantes, bem como apoiar a divulgação e o gerenciamento do acesso aos instrumentos *online* [formulário de coleta e Termo de Consentimento Livre e Esclarecido (TCLE)].

O autor principal, estudante de mestrado e homem cisgênero, atuou como coordenador da coleta de dados e participou de um treinamento de oito horas ministrado por um pesquisador em estágio pós-doutoral, com ampla experiência em pesquisa qualitativa. O treinamento foi conduzido de forma expositivo-dialogada, com caráter crítico-reflexivo, e abordou a caracterização da pesquisa qualitativa, a importância da reflexividade, bem como estratégias de abordagem e arrolamento das participantes.

À época do estudo, a equipe de pesquisa era composta por um estudante de graduação, um de mestrado, três docentes doutores e um pós-doutorando, todos integrantes de um grupo de estudos em Infecções Sexualmente Transmissíveis (IST) no contexto amazônico, com interesse em populações vulnerabilizadas. 

A coleta de dados do macroprojeto ocorreu por meio de divulgação online do formulário e, adicionalmente, por abordagens diretas a possíveis participantes. O coordenador de coleta estabeleceu contato prévio com enfermeiras para apresentar os objetivos e razões do estudo, fortalecendo a relação de confiança. Além disso, os coletadores de dados realizaram contato com profissionais de suas regiões e localidades, que, por sua vez, indicaram outras enfermeiras aptas a participar. Esse procedimento, semelhante à técnica de amostragem em bola de neve, foi adotado para ampliar o alcance do estudo e incluir participantes que poderiam não ser alcançadas apenas pela divulgação *online*.

### Tratamento e análise dos dados

O processamento dos dados qualitativos foi realizado utilizando o *software Interface de R pour les Analyses Multidimensionnelles de Textes et de Questionnaires (*IRaMuTeQ) e a análise foi discutida entre os autores. Entre as opções de análise textual, optou-se pela Classificação Hierárquica Descendente (CHD), proposta por Reinert^23^. Esta técnica classifica os segmentos de texto com base nos seus próprios vocabulários, subdividindo-os conforme a frequência das formas. Os elementos incluídos em suas respectivas classes foram aqueles com frequência ≥ 40 de ocorrências no corpus e associação com a classe determinada por um valor de χ² a ≥ 3,98, considerando o intervalo de confiança de 95% e um nível de significância de 5% (*p*< 0,05). A CHD realiza um processamento semântico e identifica o contexto de uso dos vocábulos.

Posteriormente, realizou-se análise de conteúdo com formulação temática, a partir do processamento do corpus no IRaMuTeQ por meio da CHD, que gerou a estrutura de classes e funcionou como árvore inicial para a análise temática. Esse procedimento possibilitou uma codificação inicial, ao classificar semanticamente os termos em classes, etapa que corresponde ao processo de codificação da análise de conteúdo. A seguir, as etapas de exploração do material, tratamento dos resultados, inferência e interpretação foram conduzidas à luz da análise de conteúdo temática^24^.

Por tratar-se de formulário *online* autopreenchido e anônimo, não houve devolutiva das respostas às participantes. Ademais, não foi objetivo central deste estudo alcançar saturação teórica, uma vez que se trata de recorte de um macroprojeto; entretanto, considerou-se que a amostra foi suficiente para adensar as categorias e atender ao objetivo qualitativo. Do mesmo modo, não foi realizada devolutiva das interpretações às participantes, limitando-se a análise à interpretação dos pesquisadores.

### Aspectos éticos

O projeto mais amplo que originou este artigo foi aprovado pelo Comitê de Ética em Pesquisa do Instituto de Ciências da Saúde da Universidade Federal do Pará (Parecer n.º 5.966.169 de 27 de março de 2023, CAAE 66895423.0.0000.0018). A pesquisa original contemplou abordagem quantitativa e qualitativa e incluiu três objetivos específicos. O presente estudo corresponde a um recorte dessa investigação, referente ao terceiro objetivo específico, que aborda exclusivamente a etapa qualitativa, baseada na análise das respostas abertas no formulário de coleta. Todos os participantes assinaram o TCLE.

Para assegurar o anonimato, as falas foram identificadas por meio de códigos alfanuméricos. Optou-se por manter a numeração original atribuída no macroprojeto, de modo que a sequência numérica não corresponde ao total de participantes deste recorte (n=94), mas ao conjunto maior de respondentes (n=442). Essa estratégia visou preservar a padronização do banco de dados e garantir a rastreabilidade analítica, em consonância com os princípios éticos da pesquisa qualitativa e as recomendações do COREQ^20^.

## Resultados

Adotou-se, ao longo do texto, o uso do termo enfermeira no feminino como opção terminológica, sendo que, entre as 94 participantes, 79 (84,0%) se autodeclararam do gênero feminino. Entre elas, 48 (51,0%) tinham idade superior a 35 anos. As respondentes estavam distribuídas nas cinco regiões do Brasil: Norte (n=37; 39,4%), Nordeste (n=15; 16,0%), Centro-Oeste (n=seis; 6,4%), Sul (n=10; 10,6%) e Sudeste (n=26; 27,6%).

As enfermeiras possuíam especializações em Saúde Pública relacionada com a APS (n=52, 55,3%) ou Mestrado/Doutorado (n=17, 18,1%). A maioria nunca recebeu treinamento de cuidados específicos para mulheres transexuais (n=52, 55,3%) e não adotava protocolos de atendimento para mulheres transexuais em seu serviço de saúde (n=75, 79,8%). 

Em relação aos aspectos profissionais, concluíram a Graduação em Enfermagem há menos de 10 anos (n=60, 63,8%), como também atuavam na ESF há menos de 10 anos (n=66, 70,2%), possuíam apenas um vínculo empregatício (n=71, 77,6%) e ganhavam menos de cinco salários-mínimos (salário-mínimo no Brasil em 2023: R$ 1.320,00) (n=61, 64,9%). 

O processamento do *corpus* (54 páginas), com a realização da CHD, denotou 6.603 ocorrências de termos, apresentadas em 1.478 formas distintas, com 94 segmentos de textos, considerando cada parágrafo do *corpus* como um único segmento de texto. A análise reteve 75,5% de aproveitamento do *corpus*, considerado satisfatório. O objetivo desse processamento foi identificar indicadores de significados nas respostas dos participantes. A CHD gerou quatro classes semânticas (Figura 1) e agrupou os termos associados entre si e utilizados pelos participantes para atribuir significado aos objetos em discussão.

**Figura 1:** Classes integrantes do dendrograma gerado pela classificação hierárquica descendente no *software* IRaMuTeQ. Belém, PA, Brasil

*Corpus*	Classes e temas	Postulados da Teoria do Cuidado Transcultural	Termos (f*/x^2†^)
**“Enfermeiras_mulheres-trans_ESF”**	**Classe 1**	Diversidade e Universalidade do Cuidado	Transgênero (100/14,57), população (50/13,77), compreender (100/10,77), Atenção primária à saúde (50/7,54), capacitação (50/7,74), respeitar (66,67/7,31), dificuldade (46,15/5,09), preconceito (40/4,86), rede (83,33/16,75), qualificado (60/4,32), falta (66,67/5,51).
	Desafios e barreiras no atendimento de saúde	Barreira Cultural e Acessibilidade	
	**Classe 2**	Congruência Cultural no Cuidado	Grupo (77,78/21,95), prestar (70/18,59), ação (83,33/16,75), público (44/14,37), cuidado (44,44/9,31), promover (75/8,18), necessidade (60,67/4,36), ofertar (60/5,53), procedimento (66,67/4,36), conscientização (66,67/4,36), assistência (44,44/3,98).
	Ações e cuidados essenciais na Atenção Primária à Saúde		
	**Classe 3**	Ação e Decisão de Cuidados Culturalmente Competentes	Usuário (77,78/11,5), distinção (100/10,09), ambulatório (80,33/9,09), atendimento (45,71/8,63), respeito (100/7,46), PrEP^‡^ (80/6,57), PEP^§^ (75/4,2), treinamento (60/5,17), mulher (62,5/4,69), inclusão (66,67/4,33).
	Inclusão e respeito no atendimento		
	**Classe 4**	Competência Cultural	agente comunitário de saúde (90/21,99), médico (100/13,72), trazer (100/13,72), visita (100/7,99), domiciliar (100/7,99), aceitar (100/7,99), enfermagem (71,43/7,18), ativo (80/7,14), consulta (62,5/5,25), paciente (53,85/5,19), principal (66,67/4,80), independente (75/4,59).
	Papel dos profissionais de saúde e do Agente Comunitário de Saúde		

*f = Frequência, ^†^x^2^ = Valor do teste qui-quadrado, ^‡^PrEP = Profilaxia Pré-exposição, ^§^PEP = Profilaxia Pós-exposição

As classes foram organizadas em categorias temáticas e interpretadas de acordo com os conceitos da Teoria do Cuidado Transcultural de Leininger^22^.

### Classe temática 1 - Desafios e Barreiras no Atendimento de Saúde

A diversidade e universalidade do cuidado são refletidas na necessidade de adaptação dos serviços de saúde para atender às diferentes necessidades das mulheres transexuais. A universalidade do cuidado é reforçada pela empatia e pelo acolhimento, enquanto a diversidade se manifesta nas barreiras específicas enfrentadas, como preconceito e estigma. *Por puro preconceito. Não é abordado essa temática pelas referências técnicas de saúde da mulher. [...] A APS é a porta de entrada para acolhimento e prevenção à saúde* (Enf_12)*. Preconceito, falta de informação, despreparo dos profissionais de saúde no relacionamento com pessoas transgêneros* (Enf_45). *Estigma por parte de outros profissionais que atuam na unidade, de como abordar esse público e oferecer acolhimento adequado* (Enf_39). *Preconceito, falta de informação, despreparo dos profissionais de saúde no relacionamento com pessoas transgêneros* (Enf_404). *O principal desafio é a mulher transgênero se aceitar; elas normalmente sofrem muito preconceito e devido a isso dificultam no atendimento* (Enf_282).

As barreiras culturais e a acessibilidade são destacadas pelos desafios enfrentados no atendimento. A acessibilidade é comprometida pela falta de preparo dos profissionais e pela ausência de protocolos específicos. *Falta de treinamento e fluxo definido para atendimento às pessoas transgêneros* (Enf_92). *Não há treinamento para o atendimento a essas mulheres transgêneros, desconhecimento dos fluxos de atendimento [...]* (Enf_76). *Não observo protocolo clínico para direcionar o atendimento* (Enf_45). *A falta de conhecimento sobre a rede que poderia auxiliar em alguns processos a essas pacientes, a falta de capacitação técnica no atendimento a esse público [...]* (Enf_204).

### Classe temática 2 - Ações e Cuidados Essenciais na Atenção Primária à Saúde 

A congruência cultural no cuidado é refletida na oferta de serviços e na promoção de um ambiente acolhedor e empático. A prática de escuta ativa e a personalização do atendimento são fundamentais para garantir que os cuidados sejam culturalmente congruentes. *Oferto atividade em grupo, realizo exames, e peço para retornar. Eu busco transparecer empatia, humanização e orientação sobre os cuidados em saúde nos seus mais diversos aspectos, sendo físico, psicológico, biológico e social* (Enf_107). *Acolhimento, escuta efetiva, vínculo, oferta de serviços em nível primário e em todos os serviços* (Enf_282)*. Realizo atendimento de forma integral, sem distinção* (Enf_421*). Sou responsável pela linha de IST, pertenço à comunidade LGBT* [Lésbicas, Gays, Bissexuais e Transgêneros]*, escuto com empatia, faço a anamnese completa, quebro o gelo e o estigma, promovo um local de acolhimento e oferto todos os serviços disponíveis* (Enf_305*).*

### Classe temática 3 - Inclusão e Respeito no Atendimento

Destacou-se a ausência de capacitação dos profissionais de saúde como um desafio central para o combate ao preconceito e melhoria da qualidade do atendimento às mulheres transexuais. *Falta de capacitação sobre como atender esse público* (Enf_09). *Falta de treinamento frente ao atendimento de mulheres transgêneros, dificuldade dessa população em usufruir de assistência em saúde de qualidade e falta de profissionais qualificados e humanizados para atender [...]* (Enf_25). *Falta de incentivo da gestão, falta de capacitação, dificuldade de adesão das mulheres transgêneros nas atividades da unidade. Possuo perfil profissional apropriado, sou um homem cis gay, tenho qualificação profissional, habilidades e vivências em meu cotidiano com a problemática* (Enf_30). *Capacitação técnica e oferta de serviços tendo em vista o acolhimento do paciente* (Enf_21). *Capacitação constante para evitar preconceitos e melhorar o atendimento* (Enf_34). *Precisamos de treinamento especializado para aprender a melhor forma de acolher e abordar as mulheres transgêneros. Ter um fluxo de atendimento específico que possamos utilizar no acompanhamento dessa demanda (*Enf_194*). Que as mulheres transgêneros cheguem à APS com maior frequência, que os profissionais compreendam e respeitem, que aumentem treinamentos para atendimento [...] e que seja criado um protocolo para atendimento sem barreiras* (Enf_384*).*

Porém, a ação e a decisão de cuidados culturalmente competentes são visíveis na inclusão de práticas específicas, como o uso de PrEP e PEP, e no treinamento contínuo dos profissionais. A competência cultural é essencial para oferecer um atendimento respeitoso e inclusivo. *Qualificação, acesso a PrEP e PEP, material para coleta de HPV* [Papilomavírus Humano] *específico, sala de espera interna* (Enf_300). *Falta de treinamento e fluxo definido para atendimento às pessoas transgênero* (Enf_457). *Todos os profissionais precisam de treinamento voltado para pessoas transgêneros* (Enf_425).

### Classe temática 4 - Papel dos Profissionais de Saúde e Agente Comunitário de Saúde

A competência cultural é evidenciada na importância do ACS e dos demais profissionais de saúde para estabelecer vínculos e cuidados comunitários adequados. *Após muito trabalho, temos contato e visita domiciliar do ACS, enfermeira ou médico, temos palestras nas escolas e na ESF, para que possamos estar mais próximos* (Enf_45). *Iniciar o atendimento na APS por intermédio da ESF torna o acesso mais facilitado até mesmo por poder contar com o trabalho dos ACS, que atuam identificando os indivíduos que estão afastados do serviço de saúde, possibilitando assim, busca ativa e a inserção deles nos atendimentos de saúde* (Enf_70). *Quantitativo adequado de ACS para trazer essas pacientes para a unidade* (Enf_19).

A disposição das enfermeiras para aprender e se adaptar às necessidades das mulheres transexuais demonstra ações de cuidados culturalmente competentes. *Respeitar a identidade de gênero e usar os pronomes corretos no atendimento* (Enf_1). *Adaptação dos protocolos para serem mais inclusivos* (Enf_28). *O ACS é a nossa ponte, assim que identifica realiza a abordagem humanizada trazendo essa mulher para a unidade onde se realiza a consulta de enfermagem (*Enf_101*).*

## Discussão

Esta pesquisa buscou explorar o cuidado transcultural prestado por enfermeiras da APS para mulheres transexuais, sobretudo, com ênfase na atuação no âmbito da ESF. Essa população possui necessidades de saúde específicas, resultantes da cultura que desenvolvem enquanto grupo identitário e das violências heterocisnormativas que enfrentam por questionarem as normas de gênero impostas. A Teoria do Cuidado Transcultural de Leininger^22^ orientou a interpretação dos achados ao desvelar a coexistência entre diversidade e universalidade do cuidado, a busca por congruência cultural na personalização das ações e a centralidade da competência cultural para sustentar práticas inclusivas. 

A universalidade do cuidado na APS constitui princípio fundamental para atender às necessidades das mulheres transexuais. No entanto, persistem desafios que comprometem a efetivação desse princípio, como a insuficiência de capacitação e o preconceito entre os profissionais de saúde, que comprometem a prática do cuidado transcultural. Os apontamentos das enfermeiras participantes mostram ainda a ausência de informações e protocolos específicos, resultando em abordagens inadequadas que perpetuam estigmas e dificultam o acesso a serviços de saúde^25^ ^)-(^ ^26^.

No plano das necessidades específicas, destacam-se demandas de hormonioterapia, acessos às profilaxias PrEP/PEP, materiais e fluxos adequados para coleta de exames, uso de nome social e reconhecimento da identidade de gênero, além de um ambiente de acolhimento com escuta qualificada. A ausência de preparo técnico e de diretrizes claras para essas frentes foi reiterada, revelando lacunas de educação permanente e de suporte institucional^27^.

Os achados permitem inferir que tais lacunas não são somente técnicas: elas expressam desigualdades estruturais de gênero que atravessam organizações de saúde. Superá-las requer, além de qualificações pontuais, mudanças organizacionais e culturais mais amplas, como gestão comprometida, responsabilização e monitoramento. A ausência de apoio institucional e de incentivos à qualificação profissional resulta em baixa adesão das mulheres transexuais às atividades das unidades de saúde, indicando a necessidade urgente de um comprometimento das lideranças em promover formação e sensibilização para transformar esses desafios em oportunidades de melhoria.

Apesar dessas barreiras, a competência cultural se apresenta como um pilar essencial na prática de cuidados. A inclusão de práticas específicas, como o uso de PrEP e PEP, bem como a capacitação contínua dos profissionais, é fundamental para oferecer um atendimento que respeite e inclua as especificidades das mulheres transgênero^12^ ^,^ ^28^. Além disso, pesquisa realizada apontou que a personalização do atendimento, por meio de escuta ativa e acolhimento, é crucial para garantir que as mulheres transexuais se sintam seguras e respeitadas durante o atendimento, promovendo uma experiência positiva na busca por cuidados^29^.

O caráter nacional do estudo, contemplando diferentes regiões, revela também potenciais assimetrias regionais no cuidado transcultural. Enquanto em algumas localidades emergem práticas de acolhimento e inclusão, em outras persistem barreiras acentuadas de acesso e despreparo profissional, refletindo coberturas desiguais da ESF e contextos culturais distintos. Essa heterogeneidade confirma o desafio de pensar o cuidado transcultural em um país multicultural, ajustando intervenções às realidades locais.

A formação de vínculos entre os profissionais de saúde e a comunidade é vista como um fator determinante para o sucesso dos cuidados prestados. A atuação dos ACS é particularmente enfatizada, pois eles estão na linha de frente do contato comunitário, desempenhando um papel vital na identificação e inclusão de pessoas que possam estar afastadas dos serviços de saúde^30^ ^)-(^ ^31^. Pesquisa^32^ apontou que estratégias como visitas domiciliares e palestras em escolas são eficazes para aproximar os serviços de saúde da comunidade, tornando o atendimento mais acessível e humanizado.

A prática de enfermagem deve ser fundamentada na empatia, acolhimento e personalização do atendimento, reconhecendo e valorizando as especificidades das mulheres transexuais. A oferta de serviços em âmbito primário deve ser abrangente e acessível, garantindo que as pessoas tenham acesso a todos os serviços disponíveis e assegurando um atendimento de qualidade contínuo^28^ ^)-(^ ^29^ ^),(^ ^33^. Isso implica um compromisso dos profissionais de saúde em tratar todas as pessoas com dignidade e respeito, promovendo um sistema de saúde inclusivo e sensível às diversidades culturais e sociais.

A implementação de protocolos adaptados e a formação contínua dos profissionais são fundamentais para garantir a atenção integral à saúde das mulheres transexuais. A adaptação e operacionalização desses protocolos devem refletir um compromisso institucional com equidade, envolvendo liderança, gestão e engajamento dos profissionais para transformar recomendações em práticas efetivas^34^ ^)-(^ ^36^. Assim, a capacitação constante emerge como um elemento-chave para a transformação do cuidado, uma vez que prepara os profissionais para lidar com as especificidades do atendimento a essa população.

Os achados deste estudo trazem implicações relevantes para a saúde e para a enfermagem. Evidenciam a necessidade de formação contínua das enfermeiras na APS para o cuidado de mulheres transexuais, bem como de protocolos inclusivos que orientem práticas sensíveis às diversidades de gênero. Reforçam ainda o papel central das enfermeiras e dos agentes comunitários de saúde na ampliação do acesso, no fortalecimento de vínculos e na promoção de um ambiente de cuidado livre de estigmas. Ao enfatizar a competência cultural como fundamento da prática, o estudo contribui para o aprimoramento da assistência em saúde e para a construção de serviços mais equânimes e inclusivos.

Contudo, este estudo apresenta limitações metodológicas. Os achados refletem especificamente as percepções de enfermeiras da APS, sobretudo aquelas atuantes na ESF, de modo que o possível viés de seleção restringe sua transferibilidade para outros contextos de atuação da enfermagem junto a mulheres transexuais. Ademais, o recorte privilegiou as percepções dessas profissionais, sem incluir diretamente a voz das usuárias, cuja participação em futuras pesquisas poderá ampliar a compreensão sobre o cuidado na APS. Também não foram contempladas outras populações dissidentes, como homens transexuais e pessoas não binárias, que demandam estudos próprios^17^. 

Em síntese, a competência cultural e o papel ativo de profissionais da ESF, em especial, as enfermeiras, são fundamentais para o cuidado transcultural prestado a mulheres transexuais no âmbito da APS. Essas práticas não apenas melhoram o acesso aos serviços de saúde, mas também promovem um ambiente de cuidado mais inclusivo, respeitoso e efetivo, que atende às necessidades únicas dessa população. A continuidade da formação e a implementação de políticas públicas que garantam a capacitação dos profissionais são essenciais para avançar no conhecimento científico e na prática de cuidados em saúde.

## Conclusão

No recorte qualitativo com enfermeiras atuantes na ESF, os achados mostram que o cuidado a mulheres transexuais no âmbito da APS avança quando há escuta qualificada, acolhimento e personalização das ações, com mediação comunitária pelos ACS; e se fragiliza diante de estigma, ausência de protocolos e lacunas formativas, repercutindo em acesso dificultado e práticas pouco sensíveis às especificidades dessa população. Ancorada na Teoria do Cuidado Transcultural, a interpretação desvela que a competência cultural não é atributo apenas individual, mas resulta de arranjos organizacionais; sua insuficiência ajuda a explicar as barreiras observadas, inclusive com heterogeneidades regionais no âmbito da APS brasileira.

Para a prática de enfermagem, os resultados implicam educação permanente em competência cultural, adaptação e operacionalização de protocolos inclusivos (linhas de cuidado e fluxos claros; indicadores mínimos como registro do nome social e oferta/encaminhamento para PrEP/PEP), matriciamento em saúde, combate à desinformação e participação de pessoas trans na coconstrução de processos de cuidado. O fortalecimento do papel dos ACS na busca ativa e na mediação com o território, a integração com serviços especializados e o compromisso da liderança com equidade e monitoramento contínuo são caminhos viáveis no cotidiano da APS para converter recomendações em práticas efetivas, ampliando acesso, qualidade e segurança do cuidado.

## Data Availability

Todos os dados gerados ou analisados durante este estudo estão incluídos neste artigo publicado.

# Translation: Cuidado transcultural de las enfermeras proporcionado a mujeres transexuales en la atención primaria de salud: estudio cualitativo*

**DOI:** 10.1590/1518-8345.7760.4923

**Authors:** Pedro Vitor Rocha Vila Nova, Glauber Weder dos Santos Silva, Arielly de Azevedo Candido, Raíssa Millena Silva Florencio, Glenda Roberta Oliveira Naiff Ferreira, Eliã Pinheiro Botelho

**Affiliations:** 1 Universidade Federal do Pará, Belém, PA, Brasil.; 2 Becario de la Coordenação de Aperfeiçoamento de Pessoal de Nível Superior (CAPES), Brasil.; 3 Secretaria de Estado da Saúde Pública do Rio Grande do Norte, Hospital Giselda Trigueiro, Natal, RN, Brasil.; 4 Universidade Federal do Pará, Faculdade de Enfermagem, Belém, PA, Brasil.; 5 Becaria del Conselho Nacional de Desenvolvimento Científico e Tecnológico (CNPq), Brasil.

**Keywords:** Enfermería Transcultural, Personas Transgénero, Poblaciones Vulnerables, Minorías Sexuales y de Género, Atención Primaria de Salud, Estrategias de Salud Nacionales

## Abstract

**Objetivo:**

explorar la atención intercultural que las enfermeras proporcionan a mujeres transexuales en la atención primaria de salud.

**Método:**

un estudio cualitativo y descriptivo, fundamentado en la Teoría del Cuidado Transcultural de Leininger. Un total de 94 enfermeros de atención primaria de todas las regiones de Brasil participaron en el estudio, mediante un formulario autoaplicado en línea que contenía preguntas abiertas de carácter discursivo. Los datos se analizaron mediante análisis temático de contenido, con el apoyo del *software* IRaMuTeQ.

**Resultados:**

surgieron cuatro categorías temáticas: desafíos y barreras en la atención de salud; acciones y atención esenciales en la atención primaria de salud; inclusión y respeto en el servicio; Papel de los profesionales de la salud y el agente comunitario de salud.

**Conclusión:**

los resultados indican que la mayoría de las enfermeras no recibieron formación específica ni tenían protocolos para la atención de mujeres transexuales, revelando desafíos relacionados con prejuicios, accesibilidad y cualificación profesional, pero también iniciativas de bienvenida, inclusión y el trabajo de los agentes comunitarios de salud.

## Destacados

**(1)** La falta de cualificación dificulta proporcionar una atención adecuada a las mujeres transexuales.

**(2)** Las barreras culturales y los prejuicios comprometen el cuidado universal e inclusivo.

**(3)** Las enfermeras refuerzan la importancia de la escucha activa y la empatía en la atención.

**(4)** La formación continua y la provisión de PrEP y PEP son esenciales para su inclusión.

**(5)** Los Agentes Comunitarios de Salud son fundamentales para la inclusión de mujeres transexuales.

## Introducción

En el contexto de las políticas públicas brasileñas, la Atención Primaria de Salud (APS) está configurada como organizadora y coordinadora de la atención dentro del ámbito de la Red de Atención de Salud (RAS), siendo la Estrategia de Salud de la Familia (ESF) el principal modelo para su fortalecimiento y consolidación^1^.

Al considerar el equipo multiprofesional que integra la APS, la enfermera se destaca como una profesional esencial para el desarrollo de acciones de promoción de la salud, actuando directamente en el proceso de atención, gestión e implementación de los principios y directrices del Sistema Único de Salud brasileño (SUS). Entre sus atribuciones privadas se encuentran la realización de consultas de enfermería, planificación, gestión, coordinación y evaluación de las prácticas de técnicos de enfermería y Agentes Comunitarios de Salud (ACS)^2^.

En este estudio, se adoptó el término “enfermera” para destacar la feminización histórica de la profesión en la APS, reconociendo la presencia de hombres, pero enfatizando la atención sigue asociada con la idea de “naturaleza femenina”. Esta configuración refuerza los estereotipos de género, hace que el trabajo sea más precario y contribuye a la devaluación de la categoría^3^. Así, la elección terminológica refleja la predominancia de las mujeres y, al mismo tiempo, problematiza sus efectos, abriendo espacio para una reflexión crítica sobre el género en la enfermería.

La posición estratégica de la enfermera en la APS contribuye directamente a cambios en el estilo de vida de las personas, minimizando los factores de riesgo y promoviendo la capacidad de autocuidado, convirtiéndolos en protagonistas del proceso de atención^4^. En este contexto, es esencial cuidar y dar la bienvenida a poblaciones clave y vulnerables, como las mujeres transexuales. Esta población vive diariamente con diversas desigualdades en salud, como estigmas y prejuicios, violencia, dificultad para acceder a servicios de salud y otros bienes sociales. La transfobia institucional, la discriminación y la falta de respeto por el nombre social están entre las principales violaciones que sufren las mujeres transexuales^5^. Estos factores, en conjunto, contribuyen a que su salud sea descuidada^6^ ^)-(^ ^7^.

La importancia de implementar los cuidados de salud inclusivos y competentes se destaca en la promoción de un entorno acogedor y libre de prejuicios^8^. Según la Teoría del Proceso de Normalización, es necesario cualificar a los profesionales de la salud en terminología de género y prácticas de atención afirmativa para reducir las barreras de acceso y mejorar la recepción en la atención^9^. Además, se evidencian los principales obstáculos a los que se enfrentan los travestis y transexuales, como la recepción insuficiente y el rechazo personal por parte de los profesionales^10^, lo que apunta a un cambio urgente en las prácticas de salud.

En este contexto, un enfoque intercultural para la atención de las mujeres transexuales en la APS, que reconozca particularidades culturales, es esencial para las enfermeras^11^. La importancia de la educación continua y la humanización de la atención es fundamental para una práctica más inclusiva y respetuosa^12^. La promoción de la atención integrada, que tiene en cuenta las especificidades de esta población, puede contribuir significativamente a mejorar la salud y el bienestar de las mujeres transexuales, además de estar alineada con políticas de salud pública orientadas a la equidad en el acceso^13^.

La producción científica sobre la atención de enfermería, especialmente la atención intercultural, dirigida a mujeres transexuales en la APS, sigue siendo incipiente y fragmentada, con un número reducido de publicaciones en el periodo de 2008 a 2021. Estudios existentes indican que los estigmas estructurales e interpersonales, asociados con la exclusión y fragmentación de la atención de salud para travestis y transexuales, aún centrados en servicios especializados, dificultan la consolidación del modelo integral recomendado por el SUS^14^ ^)-(^ ^15^. Persisten barreras de acceso a la APS, como la falta de respeto por el nombre social, además de la concentración de servicios e investigación en grandes centros urbanos, lo que limita una visión nacional y profundiza las desigualdades regionales^14^. A esto se suma la insuficiencia de la formación inicial y continua de las enfermeras para atender las demandas de esta población de manera culturalmente competente^16^. Estos conjuntos de evidencia revelan la magnitud de los desafíos a los que se enfrentan en cuanto a las lagunas en educación, práctica y políticas de salud, justificando la relevancia del presente estudio.

Dada la escasez y fragmentación de la evidencia sobre la atención de enfermería a mujeres transexuales en la APS, es esencial describir y analizar su progreso^17^. El contexto cultural y social de estas mujeres influye directamente en las prácticas de cuidado, exigiendo sensibilidad y competencia cultural por parte de los profesionales. Aunque las políticas públicas en Brasil han buscado consolidar una red de atención orientada a la equidad, la capacidad de resolución de problemas y la atención integral^18^ ^-^ ^19^, persisten desafíos para garantizar que dichas directrices se implementen en la APS. Por tanto, reconocer esta influencia cultural y social es esencial para guiar intervenciones que refuercen la calidad y la inclusión en la atención.

En este sentido, este estudio tuvo como objetivo explorar la atención intercultural proporcionada por las enfermeras a mujeres transexuales en la atención primaria de salud.

## Método

### Tipo de estudio

Se trata de un estudio cualitativo y descriptivo, guiado por las directrices de los Criterios Consolidados para la Reporte de Investigación Cualitativa (COREQ^)(^ ^20^. Este estudio es un extracto de un macroproyecto cuantitativo-cualitativo que contó con la participación de 442 enfermeras de la APS en diferentes regiones de Brasil, centrándose exclusivamente en el análisis de datos cualitativos en este artículo. El enfoque cualitativo^21^ fue elegido porque permite explorar las experiencias personales y subjetivas de las enfermeras de la APS para comprender la atención intercultural que se brinda a las mujeres transexuales, incluyendo cómo dichas experiencias pueden influir en la práctica profesional.

La Teoría de la Atención Transcultural de Leininger^22^ se utilizó como marco teórico y guía la práctica de enfermería basándose en el reconocimiento de la diversidad y universalidad de la atención, la búsqueda de la congruencia cultural entre las intervenciones y los valores/acreedores del usuario, y el desarrollo de la competencia cultural por parte de los profesionales. Central en la teoría es el eje de Acción y Decisión de la Atención Culturalmente Competente (es decir, congruente), operada en tres modos: preservación/mantenimiento de prácticas culturales beneficiosas; adaptación/negociación para ajustar la atención a creencias/rituales; y la reestandarización/reestructuración cuando son necesarios cambios para mejorar la salud. En el estudio, el término Barrera Cultural y Accesibilidad designa obstáculos culturales/organizacionales que afectan al acceso y la continuidad de la atención en la APS. Estas dimensiones apoyaron la organización e interpretación de los hallazgos.

### Escenario del estudio

El estudio se llevó a cabo a nivel nacional, en línea, mediante un formulario autoaplicado, llegando a enfermeros que trabajan en la ESF de todas las regiones de Brasil, siguiendo los criterios de elegibilidad para la investigación, sin necesidad previa de selección de unidades de salud específicas.

En los últimos años, la ESF ha ampliado su cobertura y se ha consolidado como el principal modelo para fortalecer la APS, promoviendo la reorganización de la RAS. Debido al vasto territorio brasileño y las diferencias regionales, la cobertura de la ESF varía entre regiones, estados y municipios. La región Noreste tiene la mayor cobertura de la ESF, con un 82,0%, seguida por las regiones Centro-Oeste y Norte, ambas con un 65,0%, Sur con un 63,7% y Sureste con un 51,0%.

### Periodo

Los datos se recopilaron en el periodo entre agosto y diciembre de 2023.

### Población

La población estaba compuesta por enfermeras que trabajaban en la APS, miembros de los equipos de la ESF en todas las regiones de Brasil, que trabajaban directamente en el cuidado.

### Criterios de selección

Como criterio de inclusión, se seleccionaron participantes con al menos seis meses de experiencia en la ESF y que ya hubieran recibido una consulta de enfermería para mujeres transexuales. Se adoptó el plazo de seis meses para asegurar que los participantes tuvieran la experiencia mínima de inserción en la rutina de la APS y familiaridad con la dinámica del servicio, para poder calificar sus informes. Se excluyeron a los participantes que estaban en algún tipo de permiso laboral o de vacaciones durante el periodo de recogida de datos.

### Definición de la muestra

Para este análisis cualitativo, se seleccionaron las 94 enfermeras que trabajaron en la ESF por conveniencia, y respondieron a las preguntas abiertas del formulario online autoaplicado, cuyo tiempo medio de finalización fue de 25 minutos. Así, la muestra incluyó participantes de todas las regiones del país, sin la intención de garantizar la representatividad nacional, pero contemplando la diversidad geográfica de los encuestados.

### Instrumento de recogida de datos 

Los datos se recopilaron mediante la autoaplicación de un formulario cuantitativo-cualitativo en línea, disponible en la plataforma Google Forms, que permite solo una presentación mediante Protocolo de Internet (IP), garantizando una mayor seguridad y evitando duplicaciones. El contacto anterior y el enlace al formulario se distribuyeron a las enfermeras a través de mensajería electrónica (WhatsApp), red social (Instagram) o correo electrónico. No hubo ninguna nota de campo porque era una encuesta online.

El formulario específico del macroproyecto contenía 31 preguntas cerradas (opción múltiple) y dos preguntas abiertas de naturaleza discursiva, lo que apoyó el análisis cualitativo de este estudio. En el presente extracto, de las preguntas cerradas, se utilizaron variables únicamente para caracterizar el perfil sociodemográfico (edad, identidad de género), trabajo (tiempo de trabajo en la APS, número de contratos, tiempo desde la formación, remuneración) y perfil académico (formación y cualificación) de los participantes. Las preguntas abiertas abordaron las experiencias en el cuidado de mujeres transexuales en la APS, a saber: describe tu cuidado y la prestación de atención a mujeres transexuales en su realidad en la ESF; ¿qué desafíos y necesidades identifica en el cuidado de mujeres transgénero en la APS? Las preguntas fueron previamente evaluadas y validadas por el grupo de investigación antes de su solicitud. 

Para llevar a cabo la investigación, se seleccionaron 10 recopiladores de datos (cinco estudiantes de grado y cinco de máster) de las cinco macroregiones de Brasil, quienes recibieron formación específica para identificar e invitar a posibles participantes, así como para apoyar la difusión y gestión del acceso a instrumentos en línea [formulario de recogida y Formulario de Consentimiento Informado (FCI)].

El autor principal, un estudiante de máster y hombre cisgénero, actuó como coordinador de la recopilación de datos y participó en un curso de formación de ocho horas impartido por un investigador en una pasantía postdoctoral, con amplia experiencia en investigación cualitativa. La formación se llevó a cabo de forma expositiva y dialogada, con un carácter crítico-reflexivo, y abordó la caracterización de la investigación cualitativa, la importancia de la reflexividad, así como estrategias para acercarse e involucrar a los participantes.

En el momento del estudio, el equipo de investigación estaba compuesto por un estudiante de grado, un estudiante de máster, tres profesores de doctorado y un investigador postdoctoral, todos miembros de un grupo de estudio sobre Infecciones de Transmisión Sexual (ITS) en el contexto amazónico, con interés en poblaciones vulnerables.

La recogida de datos del macroproyecto se realizó mediante la difusión en línea del formulario y, además, mediante enfoques directos a posibles participantes. El coordinador de recogida de datos estableció un contacto previo con las enfermeras para presentar los objetivos y razones del estudio, fortaleciendo así la relación de confianza. Además, los recopiladores de datos contactaron con profesionales de sus regiones y localidades, quienes, a su vez, indicaron que otras enfermeras podían participar. Este procedimiento, similar a la técnica de muestreo bola de nieve, se adoptó para ampliar el alcance del estudio e incluir a participantes que quizá no serían alcanzados solo mediante la difusión en línea.

### Procesamiento y análisis de datos

El procesamiento de datos cualitativos se realizó utilizando el *software Interface de R pour les Analyses Multidimensionnelles de Textes et de Questionnaires* (IRaMuTeQ) y el análisis fue debatido entre los autores. Entre las opciones para el análisis textual, se eligió la Clasificación Jerárquica Descendente (DHC) propuesta por Reinert^23^. Esta técnica clasifica los segmentos de texto según su propio vocabulario, subdividiéndolos según la frecuencia de las formas. Los elementos incluidos en sus respectivas clases fueron aquellos con una frecuencia ≥ 40 ocurrencias en el corpus y asociación con la clase determinada por un valor de χ² a ≥ 3,98, considerando un intervalo de confianza del 95% y un nivel de significación del 5% (p< 0,05). El CHD realiza el procesamiento semántico e identifica el contexto de uso de las palabras.

Posteriormente, se realizó un análisis de contenido con formulación temática, basado en el procesamiento del corpus en IRaMuTeQ a través de la CHD, que generó la estructura de clases y funcionó como un árbol inicial para el análisis temático. Este procedimiento permitía una codificación inicial, clasificando semánticamente los términos en clases, un paso que corresponde al proceso de codificación del análisis de contenido. A continuación, se realizaron las etapas de exploración del material, tratamiento de resultados, inferencia e interpretación a la luz del análisis temático de contenido^24^.

Como este es un formulario online autoaplicado y anónimo, no hubo comentarios sobre las respuestas a los participantes. Además, no era el objetivo principal de este estudio alcanzar la saturación teórica, ya que es un macroproyecto; sin embargo, se consideró que la muestra era suficiente para densificar las categorías y cumplir el objetivo cualitativo. Del mismo modo, no se dio retroalimentación a los participantes y el análisis se limitó a la interpretación de los investigadores.

### Aspectos éticos

El proyecto más amplio que originó este artículo fue aprobado por el Comité de Ética en Investigación del Instituto de Ciencias de la Salud de la Universidad Federal de Pará (Opinión n.º 5.966.169 de 27 de marzo de 2023, CAAE 66895423.0.0000.0018). La investigación original incluía un enfoque cuantitativo y cualitativo y tres objetivos específicos. El presente estudio corresponde a un extracto de esta investigación, que se refiere al tercer objetivo específico, que aborda exclusivamente la etapa cualitativa, basándose en el análisis de las respuestas abiertas en el formulario de recopilación. Todos los participantes firmaron el FCI.

Para garantizar el anonimato, los discursos se identificaron mediante códigos alfanuméricos. Se decidió mantener la numeración original asignada en el macroproyecto, de modo que la secuencia numérica no correspondiera al número total de participantes en esta sección (n=94), sino al conjunto más amplio de encuestados (n=442). Esta estrategia tenía como objetivo preservar la estandarización de la base de datos y garantizar la trazabilidad analítica, en línea con los principios éticos de la investigación cualitativa y las recomendaciones de COREQ^20^.

## Resultados

A lo largo del texto, el término enfermera se utilizó como opción terminológica, y entre los 94 participantes, 79 (84,0%) se declararon mujeres. De ellos, 48 (51,0%) tenían más de 35 años. Los encuestados se distribuyeron en las cinco regiones de Brasil: Norte (n=37; 39,4%), Noreste (n=15; 16,0%), Medio Oeste (n=seis; 6,4%), Sur (n=10; 10,6%) y Sureste (n=26; 27,6%).

Las enfermeras tenían especializaciones en Salud Pública relacionadas con PHC (n=52, 55,3%) o Máster/Doctorado (n=17, 18,1%). La mayoría nunca había recibido formación específica en atención para mujeres transexuales (n=52, 55,3%) y no adoptó protocolos de atención para mujeres transexuales en su servicio de salud (n=75, 79,8%).

En cuanto a los aspectos profesionales, completó su grado en Enfermería hace menos de 10 años (n=60, 63,8%), además de haber trabajado en la FHS menos de 10 años (n=66, 70,2%), tuvo solo un empleo (n=71, 77,6%) y ganó menos de cinco salarios mínimos (salario mínimo en Brasil en 2023: R$ 1.320,00) (n=61, 64,9%).

El procesamiento del *corpus* (54 páginas), con la realización de la CHD, denotó 6.603 ocurrencias de términos, presentadas en 1.478 formas distintas, con 94 segmentos de texto, considerando cada párrafo del corpus como un único segmento de texto. El análisis retuvo el 75,5% del uso del corpus, lo cual se consideró satisfactorio. El objetivo de este procesamiento fue identificar indicadores de significado en las respuestas de los participantes. La CHD generó cuatro clases semánticas (Figura 1) y agrupó los términos asociados entre sí y que los participantes usaron para atribuir significado a los objetos en discusión.

**Figura 1:** Clases integrantes del dendrograma generado por la clasificación jerárquica descendente en el *software* IRaMuTeQ. Belém, PA, Brasil

*Corpus*	Clases y temas	Postulados de la Teoría del Cuidado Transcultural	Términos (f*/x^2†^)
**“Enfermeras_mujeres-trans_ESF”**	**Clase 1**	Diversidad y Universalidad del Cuidado	Transgénero (100/14,57), población (50/13,77), comprender (100/10,77), Atención primaria de salud (50/7,54), capacitación (50/7,74), respetar (66,67/7,31), dificultad (46,15/5,09), prejuicio (40/4,86), red (83,33/16,75), cualificado (60/4,32), falta (66,67/5,51).
	Desafíos y barreras en la atención de salud	Barrera Cultural y Accesibilidad	
	**Clase 2**	Congruencia Cultural en el Cuidado	Grupo (77,78/21,95), prestar (70/18,59), acción (83,33/16,75), público (44/14,37), cuidado (44,44/9,31), promover (75/8,18), necesidad (60,67/4,36), ofertar (60/5,53), procedimiento (66,67/4,36), concienciación (66,67/4,36), asistencia (44,44/3,98).
	Acciones y cuidados esenciales en la Atención Primaria de Salud		
	**Clase 3**	Acción y Decisión de Cuidados Culturalmente Competentes	Usuario (77,78/11,5), distinción (100/10,09), ambulatorio (80,33/9,09), atención (45,71/8,63), respeto (100/7,46), PrEP^‡^ (80/6,57), PEP^§^ (75/4,2), formación (60/5,17), mujer (62,5/4,69), inclusión (66,67/4,33).
	Inclusión y respeto en la atención		
	**Clase 4**	Competencia Cultural	agente comunitario de salud (90/21,99), médico (100/13,72), traer (100/13,72), visita (100/7,99), domiciliar (100/7,99), aceptar (100/7,99), enfermería (71,43/7,18), activo (80/7,14), consulta (62,5/5,25), paciente (53,85/5,19), principal (66,67/4,80), independiente (75/4,59).
	Papel de los profesionales de salud y del Agente Comunitario de Salud		

*f = Frecuencia, ^†^x^2^ = Valor de la prueba del chi-cuadrado, ^‡^PrEP = Profilaxis Preexposición, ^§^PEP = Profilaxis Postexposición

Las clases fueron organizadas en categorías temáticas e interpretadas según los conceptos de la Teoría del Cuidado Transcultural de Leininger^22^.

### Clase temática 1 - Desafíos y Barreras en la Atención de Salud

La diversidad y universalidad del cuidado se reflejan en la necesidad de adaptar los servicios de salud para satisfacer las diferentes necesidades de las mujeres transexuales. La universalidad del cuidado se refuerza con la empatía y la acogida, mientras que la diversidad se manifiesta en las barreras específicas que se enfrentan, como los prejuicios y el estigma. *Por puro prejuicio. Este tema no se aborda con las referencias técnicas de la salud de la mujer. [...] La PHC es la puerta de entrada a la atención sanitaria y la prevención* (Enf_12)*. Prejuicio, falta de información, falta de preparación de los profesionales sanitarios en la relación con personas transgénero* (Enf_45)*. Estigma por parte de otros profesionales que trabajan en la unidad, sobre cómo acercarse a este público y ofrecer una recepción adecuada* (Enf_39)*. Prejuicio, falta de información, falta de preparación de los profesionales sanitarios en la relación con personas transgénero* (Enf_404)*. El principal desafío es que las mujeres transgénero se acepten a sí mismas; Normalmente sufren muchos prejuicios y, por ello, dificultan la atención* (Enf_282).

Las barreras culturales y la accesibilidad se ponen de manifiesto en los retos a los que se enfrentan en la asistencia. La accesibilidad se ve comprometida por la falta de preparación de profesionales y la ausencia de protocolos específicos. *A falta de conhecimento sobre a rede que poderia auxiliar em alguns processos a essas pacientes, a falta de capacitação técnica no atendimento a esse público [...]* (Enf_204). *Falta de formación y de un flujo definido para la atención a personas transgénero* (Enf_92). *No hay formación para la atención de estas mujeres transgénero, falta de conocimiento sobre los flujos de atención* [...] (Enf_76). *No sigo un protocolo clínico para dirigir la atención* (Enf_45). *La falta de conocimiento sobre la red que podría ayudar en algunos procesos para estos pacientes, la falta de formación técnica en la atención de este público* [...] (Enf_204).

### Clase temática 2 - Acciones y Cuidados Esenciales en la Atención Primaria de Salud

La congruencia cultural en el cuidado se refleja en la prestación de servicios y en la promoción de un entorno acogedor y empático. La práctica de la escucha activa y la personalización de la atención son esenciales para garantizar que los cuidados sean culturalmente coherentes. *Ofrezco actividades grupales, realizo exámenes y solicito consultas de seguimiento. Busco transmitir empatía, humanización y orientación sobre el cuidado de la salud en sus diversos aspectos: físico, psicológico, biológico y social. (*Enf_107). *Acogida, escucha eficaz, unión y prestación de servicios a nivel primario y en todos los servicios* (Enf_282)*. Ofrezco atención integral, sin distinción* (Enf_421). *Soy responsable de la línea de ITS, pertenezco a la comunidad LGBT [Lesbianas, Gays, Bisexuales y Transgéneros], escucho con empatía, hago la anamnesis completa, rompo el hielo y el estigma, promuevo un lugar acogedor y ofrezco todos los servicios disponibles* (Enf_305).

### Clase temática 3 - Inclusión y respecto en la Atención

La falta de capacitación de profesionales de la salud se destacó como un desafío central para combatir los prejuicios y mejorar la calidad de la atención a las mujeres transexuales. *Falta de capacitación sobre cómo servir a este público* (Enf_09). *La falta de formación en la atención a mujeres transgénero, la dificultad de esta población para disfrutar de una atención de salud de calidad y la falta de profesionales cualificados y humanizados para ayudar [...]* (Enf_25)*. Falta de incentivos por parte de la dirección, falta de capacitación, dificultad para que las mujeres transgénero sigan las actividades de la unidad. Tengo un perfil profesional adecuado, soy un hombre cis gay, tengo una cualificación profesional, habilidades y experiencias en mi vida diaria con el problema* (Enf_30)*. Capacitación técnica y prestación de servicios con el fin de acoger al paciente* (Enf_21). *Capacitación constante para evitar prejuicios y mejorar el servicio* (Enf_34). *Necesitamos formación especializada para aprender cómo acoger y acercarnos mejor a las mujeres transgénero. Disponer de un flujo de servicio específico que podamos usar para monitorizar esta demanda* (Enf_194)*. Que las mujeres transgénero alcanzan la APS con más frecuencia, que los profesionales las comprendan y respeten, que aumenten la formación para la atención [...] y que se cree un protocolo para la atención sin barreras* (Enf_384).

Sin embargo, la acción y la decisión de los cuidados culturalmente competentes son visibles en la inclusión de prácticas específicas, como el uso de PrEP y PEP, y en la formación continua de profesionales. La competencia cultural es esencial para ofrecer una atención respetuosa e inclusiva. *Cualificación, acceso a PrEP y PEP, material para la recolección específica de VPH* [Papilomavirus Humano] *específico, sala de espera interna* (Enf_300). *Falta de formación y de un flujo definido para la atención a personas transgénero* (Enf_457). *Todos los profesionales necesitan formación dirigida a personas transgénero* (Enf_425).

### Clase temática 4 - Papel de los Profesionales de la Salud y los Agentes Comunitarios de Salud

La competencia cultural se evidencia en la importancia de la ACS y de otros profesionales de la salud para establecer vínculos y cuidados comunitarios adecuados. *Después de mucho trabajo, tenemos contacto y visitas a domicilio del ACS, enfermero o médico, tenemos conferencias en colegios y en la ESF, para estar más cerca* (Enf_45). *Iniciar la atención en la APS a través de la ESF facilita el acceso, incluso porque puede contar con el trabajo de los ACS, que trabajan identificando a las personas que están fuera del servicio de salud, facilitando así la búsqueda activa y su inserción en la atención de salud* (Enf_70). *Cantidad adecuada de ACS para traer a estas pacientes a la unidad* (Enf_19).

La disposición de las enfermeras para aprender y adaptarse a las necesidades de las mujeres transgénero demuestra acciones de cuidados culturalmente competentes. *Respetar la identidad de género y utilizar los pronombres correctos en la atención* (Enf_1). *Adaptar los protocolos para ser más inclusivos* (Enf_28). *El ACS es nuestro puente, en cuanto lo identifica, adopta el enfoque humanizado, trayendo a esta mujer a la unidad donde se realiza la consulta de enfermería (*Enf_101*).*

## Discusión

Esta investigación buscó explorar el cuidado transcultural que proporcionan las enfermeras de la APS a mujeres transexuales, especialmente con énfasis en el trabajo dentro de la ESF. Esta población tiene necesidades de salud específicas, resultado de la cultura que desarrollan como grupo identitario y de las violencias heterocisnormativas que enfrentan por cuestionar las normas de género impuestas. La Teoría del Cuidado Transcultural de Leininger^22^ guió la interpretación de los hallazgos al revelar la coexistencia entre diversidad y universalidad del cuidado, la búsqueda de la congruencia cultural en la personalización de las acciones y la centralidad de la competencia cultural para sostener prácticas inclusivas.

La universalidad del cuidado en la APS es un principio fundamental para satisfacer las necesidades de las mujeres transexuales. Sin embargo, persisten desafíos que comprometen la eficacia de este principio, como la capacitación insuficiente y los prejuicios entre los profesionales de la salud, que comprometen la práctica del cuidado transcultural. Las notas de las enfermeras participantes también muestran la ausencia de información y protocolos específicos, lo que resulta en enfoques inadecuados que perpetúan estigmas y dificultan el acceso a los servicios de salud^25^ ^)-(^ ^26^.

En cuanto a las necesidades específicas, se destacan las demandas de terapia hormonal, acceso a las profilaxis PrEP/PEP, materiales y flujos adecuados para la recogida de pruebas, uso del nombre social y reconocimiento de identidad de género, además de un entorno acogedor con escucha cualificada. Se reiteró la ausencia de preparación técnica y directrices claras para estos frentes, lo que reveló carencias en la educación continua y el apoyo institucional^27^.

Los hallazgos nos permiten inferir que estas brechas no son solo técnicas: expresan desigualdades estructurales de género que atraviesan las organizaciones de salud. Superarlas requiere, además de cualificaciones específicas, cambios organizativos y culturales más amplios, como una gestión comprometida, responsabilidad y supervisión. La ausencia de apoyo institucional e incentivos para la cualificación profesional resulta en una baja adhesión de las mujeres transexuales a las actividades de las unidades de salud, lo que indica la urgente necesidad de un compromiso por parte de los líderes para promover la formación y la concienciación que transforme estos desafíos en oportunidades de mejora.

A pesar de estas barreras, la competencia cultural es un pilar esencial en la práctica del cuidado. La inclusión de prácticas específicas, como el uso de PrEP y PEP, así como la formación continua de profesionales, es esencial para ofrecer una atención que respete e incluya las especificidades de las mujeres transgénero^12^ ^),(^ ^28^. Además, un estudio realizado indicó que la personalización del cuidado, a través de la escucha activa y la bienvenida, es crucial para garantizar que las mujeres transexuales se sientan seguras y respetadas durante el cuidado, promoviendo una experiencia positiva en la búsqueda de cuidados^29^.

La naturaleza nacional del estudio, que abarca diferentes regiones, también revela posibles asimetrías regionales en el cuidado transcultural. Mientras que en algunos lugares surgen prácticas de acogida e inclusión, en otros existen barreras acentuadas al acceso y falta de preparación profesional, reflejando coberturas desiguales de la ESF y diferentes contextos culturales. Esta heterogeneidad confirma el desafío de pensar en el cuidado transcultural en un país multicultural, ajustando las intervenciones a las realidades locales.

La formación de vínculos entre los profesionales de la salud y la comunidad se considera un factor determinante para el éxito de los cuidados proporcionados. Se enfatiza especialmente el desempeño de los ACS, ya que están en primera línea del contacto comunitario, desempeñando un papel vital en la identificación e inclusión de personas que pueden estar alejadas de los servicios de salud^30^ ^)-(^ ^31^. Un estudio^32^ indicó que estrategias como las visitas domiciliarias y las conferencias en las escuelas son efectivas para acercar los servicios de salud a la comunidad, haciendo que la atención sea más accesible y humanizada.

La práctica de enfermería debe basarse en la empatía, la acogida y la personalización del cuidado, reconociendo y valorando las especificidades de las mujeres transexuales. La prestación de servicios a nivel primario debe ser integral y accesible, asegurando que las personas tengan acceso a todos los servicios disponibles y garantizando una atención de calidad continua^28^ ^)-(^ ^29^ ^),(^ ^33^. Esto implica un compromiso por parte de los profesionales de la salud de tratar a todas las personas con dignidad y respeto, promoviendo un sistema de salud inclusivo y sensible a las diversidades culturales y sociales.

La implementación de protocolos adaptados y la formación continua de los profesionales son esenciales para garantizar una atención de salud integral a las mujeres transexuales. La adaptación y operativización de estos protocolos debe reflejar un compromiso institucional con la equidad, implicando liderazgo, gestión y compromiso de profesionales para transformar las recomendaciones en prácticas efectivas^34^ ^)-(^ ^36^. Así, la capacitación constante se convierte en un elemento clave para la transformación del cuidado, ya que prepara a los profesionales para afrontar las especificidades de la atención para esta población.

Los hallazgos de este estudio tienen implicaciones relevantes para la salud y la enfermería. Subrayan la necesidad de formación continua de enfermeras en la APS para el cuidado de mujeres transexuales, así como protocolos inclusivos que guíen prácticas sensibles a la diversidad de género. También refuerzan el papel central de las enfermeras y agentes comunitarios de salud en la ampliación del acceso, el fortalecimiento de los lazos y la promoción de un entorno de cuidado libre de estigmas. Al enfatizar la competencia cultural como base de la práctica, el estudio contribuye a la mejora de la atención de salud y a la construcción de servicios más equitativos e inclusivos.

Sin embargo, este estudio presenta limitaciones metodológicas. Los hallazgos reflejan específicamente las percepciones de las enfermeras de la APS, especialmente de las que trabajan en la ESF, de modo que el posible sesgo de selección restringe su transferibilidad a otros contextos de trabajo de enfermería con mujeres transexuales. Además, el enfoque se centró en las percepciones de estas profesionales, sin incluir directamente la voz de las usuarias, cuya participación en futuras investigaciones podría ampliar la comprensión del cuidado en la APS. Otras poblaciones disidentes, como hombres transexuales y personas no binarias, que requieren sus propios estudios, tampoco fueron incluidas^17^.

En resumen, la competencia cultural y el papel activo de los profesionales de la ESF, especialmente las enfermeras, son fundamentales para el cuidado transcultural que se ofrece a las mujeres transexuales en el contexto de la APS. Estas prácticas no solo mejoran el acceso a los servicios de salud, sino que también promueven un entorno de cuidado más inclusivo, respetuoso y eficaz que satisfaga las necesidades únicas de esta población. La continuidad de la formación y la implementación de políticas públicas que garanticen la capacitación de profesionales son esenciales para avanzar en el conocimiento científico y la práctica de cuidados de salud.

## Conclusión

En el corte cualitativo con enfermeras que trabajan en la ESF, los hallazgos muestran que el cuidado a las mujeres transexuales en el entorno de la APS avanza cuando hay escucha cualificada, acogida y personalización de las acciones, con mediación comunitaria por parte de los ACS; y se debilita ante el estigma, la ausencia de protocolos y las lagunas formativas, lo que resulta en un acceso difícil y prácticas poco sensibles a las especificidades de esta población. Anclada en la Teoría del Cuidado Transcultural, la interpretación revela que la competencia cultural no es solo un atributo individual, sino que resulta de los arreglos organizativos; su insuficiencia ayuda a explicar las barreras observadas, incluidas las heterogeneidades regionales dentro del ámbito de la APS brasileña.

Para la práctica de enfermería, los resultados implican educación continua en competencia cultural, adaptación y operativización de protocolos inclusivos (líneas claras de cuidado y flujos; indicadores mínimos como el registro del nombre social y oferta/derivación a PrEP/PEP), apoyo matricial en salud, combate a la desinformación y participación de personas trans en la coconstrucción de procesos de atención. Fortalecer el papel de los ACS en la búsqueda activa y mediación con el territorio, la integración con servicios especializados y el compromiso de la dirección con la equidad y la supervisión continua son vías viables en la rutina diaria de la APS para convertir recomendaciones en prácticas efectivas, ampliando el acceso, la calidad y la seguridad del cuidado.

## Data Availability

Todos los datos generados o analizados durante este estudio están incluidos en este artículo publicado.
